# Occurrence of Side Effects in Treatment-Resistant Depression: Role of Clinical, Socio-Demographic and Environmental Characteristics

**DOI:** 10.3389/fpsyt.2021.795666

**Published:** 2021-12-06

**Authors:** Anna Levy, Wissam El-Hage, Djamila Bennabi, Etienne Allauze, Alexandra Bouvard, Vincent Camus, Philippe Courtet, Jean-Michel Dorey, Bruno Etain, Guillaume Fond, Jean-Baptiste Genty, Jérôme Holtzmann, Mathilde Horn, Marion Leboyer, Pierre-Michel Llorca, Manon Meyrel, Fanny Molière, Anne-Sophie Nguon, Jean Petrucci, Romain Rey, Raphaelle Richieri, Florian Stephan, Guillaume Vaiva, Michel Walter, Emmanuel Haffen, Bruno Aouizerate, Antoine Yrondi

**Affiliations:** ^1^Fondation FondaMental, Créteil, France; ^2^Service de Psychiatrie et de Psychologie Médicale de l'adulte (Department of Psychiatry and Adult Medical Psychology), Centre Expert Dépression Résistante FondaMental (FondaMental Advanced Centre of Expertise in Resistant Depression, CHU de Toulouse (University Hospital Centre), Hôpital Purpan, ToNIC Toulouse NeuroImaging Centre, Université de Toulouse (Toulouse University), INSERM, UPS, Toulouse, France; ^3^U1253, iBrain, CIC1415, Inserm, CHRU de Tours (Regional University Hospital Centre), Université de Tours, Tours, France; ^4^Service de Psychiatrie, Centre Expert Dépression Résistante FondaMental, CIC-1431 INSERM, CHU de Besançon, EA 481 Neurosciences, Université de Bourgogne Franche Comté, Besançon, France; ^5^Department of Psychiatry, CHU Clermont-Ferrand, University of Clermont Auvergne, EA 7280, Clermont-Ferrand, France; ^6^Pôle de Psychiatrie Générale et Universitaire (Department of General and University Academic Psychiatry Cluster), Centre de référence régional des pathologies anxieuses et de la dépression (Regional Reference Center for the Management and Treatment of Anxiety and Depressive Disorders), Centre Expert Dépression Résistante FondaMental (FondaMental Advanced Centre of Expertise in Resistant Depression), CH Charles Perrens, Bordeaux, Laboratoire Nutrition et Neurobiologie intégrée (Integrated Nutrition and Neurobiology Laboratory) (UMR INRAE 1286), Université de Bordeaux (Bordeaux University), Bordeaux, France; ^7^Department of Emergency Psychiatry and Acute Care, CHU Montpellier, INSERM U1061, Montpellier University, Montpellier, France; ^8^INSERM U1028, CNRS UMR5292, University Lyon 1, Lyon Neuroscience Research Centre, Psychiatric Disorders: from Resistance to Response ΨR2 Team, Centre Hospitalier Le Vinatier (Hospital Centre), Bron, France; ^9^Université de Paris et AP-HP - GHU Lariboisière-Fernand Widal - Département de Psychiatrie et de Médecine Addictologique, Paris, France; ^10^Pôle Psychiatrie, Centre Expert Dépression Résistante FondaMental, CHU La Conception, Marseille, France; ^11^Université Paris-Est, UMR_S955, UPEC, Créteil, France Inserm, U955, Equipe 15 Psychiatrie génétique (Team 15 Genetic Psychiatry), Créteil, France AP-HP, Hôpital H. Mondor-A. Chenevier, Pôle de psychiatrie (Psychiatry Cluster), Créteil, France Fondation FondaMental, Fondation de Cooperation Scientifique (Scientific Cooperation Foundation), Créteil, France; ^12^Service Hospitalo-Universitaire de Psychiatrie, CHU Grenoble Alpes, Université Grenoble Alpes, Inserm, U1216, CHU Grenoble Alpes, Grenoble Institut Neurosciences (Institute of Neurosciences), Grenoble, France; ^13^Service de Psychiatrie adulte (Department of Adult Psychiatry), Centre Expert Dépression Résistante FondaMental, CHRU de Lille, Hôpital Fontan 1, Lille, France; ^14^Service Hospitalo-Universitaire de Psychiatrie Générale et de Réhabilitation Psycho Sociale 29G01 et 29G02 (University Hospital Department of General Psychiatry and Psychosocial Rehabilitation), Centre Expert Depression Résistante FondaMental, EA 7479, CHRU de Brest, Hôpital de Bohars, Brest, France; ^15^Centre National de Ressources et Résilience pour les psychotraumatismes (Cn2r Lille Paris), Lille, France

**Keywords:** expert centres, treatment-resistant depression, antidepressants, side effects, clinical severity, childhood trauma

## Abstract

**Introduction:** Treatment-resistant depression (TRD) is a disabling psychiatric condition characterized by the failure of two antidepressants (ADs). Since the occurrence of side effects (SEs) appears to be one of the main determinants of early discontinuation of pharmacological treatments contributing to a pseudo-resistance, the purpose of this study was to determine the parameters associated with the occurrence of SEs under ADs in a cohort of patients with TRD.

**Methods:** An observational, cross-sectional, multicentre study was carried out using data from the French network of Expert Centers for TRD. For the 108 patients enrolled in the study, the statistical analyses focused on the overall occurrence and on the profile of the SEs (9 categories, 32 items).

**Results:** SEs were influenced by age and sex and were positively associated with the intensity of anxious, depressive and suicidal symptoms, a history of childhood trauma (sexual abuse, emotional abuse and neglect), and negatively associated with self-esteem, and assessment of overall functioning.

**Conclusion:** Using variables accessible in common practice, these results fall within the dynamic of a more tailored approach to medicine that could allow, through integrated pharmacological management, the continuation of antidepressant treatments, and therefore limit the risk of therapeutic failure.

## Introduction

Despite the lack of global consensus defining Treatment-Resistant Depression (TRD), 60% of patients treated with ADs will not achieve remission after first-line therapy and 30% will be considered “resistant” (1–3) to any pharmacological class (4), with appropriate dosage and duration (5). In order to acknowledge treatment failure, the adequacy of the indication, the current dosage and duration of treatment must also be ensured, i.e., the relevance of the diagnosis of depression and the quality of treatment compliance (6) must be carefully considered. Although compliance with the recommended dosage is generally advocated, it nevertheless seems necessary for some ADs to reach a maximum dosage before concluding that the molecule is inefficient (7), or even for other ADs to achieve a specific plasma concentration (i.e., within the therapeutic range) because of significant inter- and intra-individual variabilities (8).

After the perceived inefficacy of the molecule, the occurrence of side effects (SEs) is one of many influential factors along with the lack of psychoeducation (9–11) precipitating early treatment interruption (12, 13). Although generally underreported and poorly researched or even assessed in trials with multiple methodological biases (short-term use in highly-selected subjects) (14), their prevalence was estimated, depending on their nature, in up to one-third of patients receiving ADs (15). In fact, even if, for some patients, the *fear* of SEs seems to be more akin with non-compliance than their actual *occurrence* (10), the mean length of time to treatment discontinuation was documented in an observational study. A difference of 28 days was highlighted between poorly-complying patients who did not experience any SE (43 days) and those who experienced SEs while receiving ADs (15 days) (16).

Based on similar observations, a Dutch study in patients with non-TRD mainly focused on the nature, prevalence and contributors of the SEs perceived by patients during the long-term use of ADs in ecological conditions (17). The authors found different profiles of SEs depending on the pharmacological classes of ADs, and a higher number of SEs reported by younger patients, with more severe depression, a greater number of concomitant psychiatric diagnoses, a higher dosage and a shorter duration of use. However, the few available results concern patients with “ordinary” depression, and the lack of evidence concerning the particular case of TRD highlights the need for more specific studies, designed for and carried out in such pathological populations.

Other potential determinants have also been proposed. The data for these can sometimes be conflicting—such as the level of anxiety associated with major depressive episodes (MDEs) (18, 19)—or even limited to the risk of poor therapeutic response (20–22). These include suicidal intensity or number of previous hospitalisations, but also previous history of childhood trauma (23) known to be associated with TRD. Therefore, particular attention should be paid to these variables and the potential contribution in the occurrence of SEs.

The main purpose of this study was therefore to assess the role of different factors mainly referring to clinical, pharmacological, sociodemographic and environmental characteristics—positively or negatively associated with the occurrence of SEs in a cohort of patients with TRD receiving various classes of ADs.

## Methods

### Patients

All the subjects enrolled in the study were referred by their general practitioner or attending psychiatrist to one of the centers of the French Network of Expert Centers for Treatment Resistant Depression (i.e., 13 specialized healthcare centers throughout France). A psychiatrist ensured that the criteria of TRD were met and conducted a full assessment before subjects were enrolled in the longitudinal cohort study.

#### Inclusion Criteria

Patients were diagnosed with TRD according to the DSM-IV criteria, and underwent an assessment of depressive symptom intensity [moderate to severe, MADRS ≥ 20 (24)] and level of resistance [Thase and Rush staging model, ≥stage 2: failure of at least 2 adequate trials of 2 distinctly different of antidepressants (25)]. The subjects were informed of the assessment protocol sequence and gave their informed consent.

#### Exclusion Criteria

Patients diagnosed with bipolar disorder, psychotic disorder, obsessive-compulsive disorder, eating disorder with low body mass index (BMI < 15), somatoform disorders and psychoactive substance-induced mood disorder.

### Data

The shared electronic medical record (e-Resistant Depression) brings together a set of standardized assessment tools. As part of this study, particular attention was paid to the data collected at baseline (visit V0).

#### Treatments

Previous and current prescriptions of ADs (name and daily dose) were described (ATHF), the data re-coded, and the patients divided into 7 groups according to their pharmacological class of AD: (1) Selective Serotonin Reuptake Inhibitors (SSRI), (2) Serotonin-Norepinephrine Reuptake Inhibitors (SNRIs), (3) Tricyclic ADs (TCAs), (4) α2 Adrenoceptor Antagonists (NASSAs, from: Noradrenergic and specific serotonergic antidepressants), (5) Monoamine Oxidase Inhibitors (MAOIs), (6) Other ADs and (7) Combination of at least 2 ADs. We stratified antidepressants according to their pharmacological classes in order to provide sufficient sample size for statistical analyses.

Subgroups were also created inside these groups, based on the therapeutic strategy extended to the use of potentiators as a result of which up to 6 categories could be defined for each class of AD: (1) Non-potentiated, (2) With Lithium, (3) With Mood Stabilizer or Antiepileptic Drug (MS/AD), (4) With Second-Generation (or atypical) Antipsychotic (SGA), (5) With another potentiator, (6) In association with at least 2 potentiators.

#### Side Effects

The SEs were collected using the modified PRISE scale (Patient-Rated Inventory of Side Effects Modified, PRISE-M). It was used to identify these SEs and assess the level of tolerance (0: absent, 1: tolerable, 2: painful) for each symptom experienced during the last 7 days in the different domains explored, i.e., gastro-intestinal, cardiovascular, skin, nervous, sensory (eyes and ears) and urogenital systems, but also: sleep sexual function, and other SEs (see Table 1). The data was re-coded according to a binary system (0: absent, 1: present) for each symptom to define an SE score evaluating their onset in terms of occurrences, regardless of their perceived severity.

**Table 1:** The 9 domains and 32 items of the PRISE-M scale.

**Domains**	**Items**
Gastrointestinal tract	Diarrhea
	Constipation
	Dry mouth (xerostomia)
	Nausea and vomiting
Cardiovascular system	Palpitations
	Vertigo
	Chest discomfort or pain
Skin system	Sweating
	Itching
	Dry skin (xeroderma)
Nervous system	Headache
	Tremor
	Impaired motor control
	Dizziness
Sensory system	Blurred vision
	Tinnitus
Urogenital system	Urines
	Painful urination
	Frequent urination
	Irregular periods
Sleep	Trouble falling asleep
	Excessive sleepiness
Sexual function	Reduced sex drive
	Orgasm disorders
	Erectile dysfunction
Other SEs	Anxiety
	Impaired concentration
	General malaise
	Agitation
	Asthenia
	Loss of energy
	Weight gain

#### Psychiatric Status

The psychiatric status was assessed using the history of past and current MDEs with the Mini International Neuropsychiatric Interview (MINI 5.0) including the number of episodes, the duration of current episode, treatment-resistant episodes and hospitalisations, age at onset, intensity of self-rated (Quick Inventory of Depressive Symptomatology, QIDS) or hetero-assessed (Montgomery-Åsberg Depression Rating Scale, MADRS) depressive symptoms, level of treatment resistance (ATHF), level of self-rated (State-Trait Anxiety Inventory, YA form, STAI-YA) or hetero-assessed (Tyrer Brief Anxiety Scale, BAS) anxiety, suicidal tendencies (Measure of Suicidal Ideation, MSI), or other factors such as self-esteem (Rosenberg scale).

#### Patient and Environment-Related Characteristics

The factors studied included age, sex, educational level, level of functioning (Global Assessment of Functioning, GAF), exposure to childhood trauma (Child Trauma Questionnaire, CTQ—specifically exploring 5 or subscores: emotional, physical and sexual abuse, emotional and physical neglect), lifestyle habits through the body mass index (BMI) and associated consumption (qualitative smoking status and estimated “pack-years”).

#### Declaration of Ethics

The database has been regulated and approved by an Ethics Committee (CNIL DR-2015–673). All the patients from the Expert Centers received oral and written information about the potential use of their data for clinical monitoring, treatment and research purposes. All the materials used for the patient assessment has been validated in French.

### Statistical Analyses

The statistical processing of the recoded data was performed using Excel® and Jamovi® (version 1.0). The descriptive statistics applied to the sample used numerical values and percentages for the discrete and categorical variables, and mean values and standard deviations for continuous variables.

Pearson correlation matrices including relevant parameters (according to evidence-based literature data) were then carried out for exploratory purposes in order to highlight the possible interaction between variables and the onset of SEs. Correlations were considered significant at the 0.05 level (two-tailed). Linear regression models were then proposed to explore relationships between the variables of interest identified (independent variables) and the SE-related data (dependent variables). Their effects were corrected using selected socio-demographic and psychometric parameters (age, sex, hetero-assessed symptomatic intensity). The level of significance for the type I error was set to 0.05. In the same way, parametric Student's tests (*t*-tests) and analyses of variance (ANOVAs) were conducted to study the categorical variables. Analyses of covariance (ANCOVAs) were also used to adjust and correct the results according to the previous parameters.

In both cases, the analyses focused on overall occurrence and on the profile of SEs using the 9-category (or domain) side-effect scale on the one hand, and the entire 32 individualized items on the other hand. At each step, the Benjamini-Hochberg false discovery rate (FDR, set at 0.05) was applied, and the *p-values* of the *post-hoc* tests were corrected using the appropriate method (Tukey or Bonferroni correction) after verification of the homogeneity of variances (Levene's test) in order to check the robustness of the analyses. Consequently, the values reported in this study correspond to the results obtained after making the appropriate corrections, as required.

## Results

### Sample

**Figure 1:**
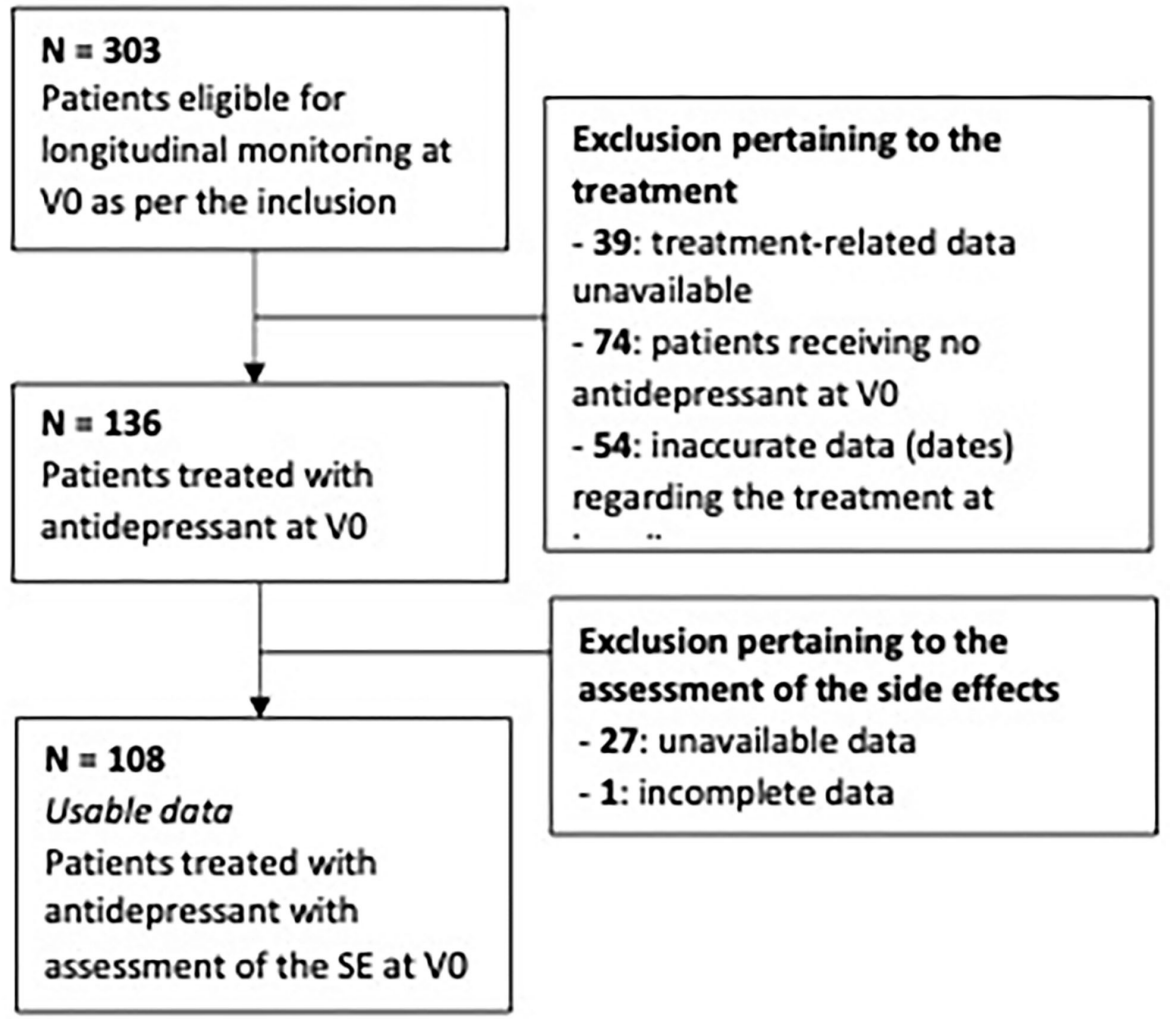
Flowchart.

The data provided by the Expert Centers included the variables that were collected at baseline from 303 patients. Among these, treatment-related information was unavailable for 39 subjects and incomplete in 54 cases, while 74 patients received no documented AD at V0. SE-related data were also unavailable for 27 subjects and incomplete in 1 case. After these individuals and further 1 duplicate, the sample therefore consisted of a set of usable data for 108 patients with at least one AD and an assessment of the SEs at V0 (Figure 1).

### Socio-Demographic and Psychopathological Characteristics

The main socio-demographic and psycho-pathological characteristics of the participants are presented in Table 2. The sample included 108 subjects, mainly women, with a mean age of 53.1 years and a mean level of education of 14.6 years from first grade. Patients with documented clinical characteristics reported an average of four MDEs prior to the current episode. The first MDE was diagnosed at a mean age of 40.6 years and only 1.52 episodes were unresponsive to at least 2 consecutive ADs. According to DSM-IV criteria, the intensity of the current episode was considered mild in 1.8% of cases, moderate in 17.9% of cases, and severe in 80.4% of cases. The latter category included severe MDEs without psychotic features (PF) (64%) as well as severe psychotic MDEs with mood-congruent (14.3%) and mood-incongruent PFs (1.8%). Clinically, 83.9% of MDEs had no other specifiers whereas 14.3% of the patients showed melancholic symptoms and 1.8% displayed atypical features. The duration of the current episode exceeded 2 years in 53.6% of cases, meeting the definition of “chronic” MDE, and the median time before introducing an AD was 4 (range = 0–364) weeks.

**Table 2:** Socio-demographic and psychopathological characteristics of the participants.

**Sex**	***N =* 108**
Female/Male	67/41
Percentage of Females	62
**Age**	***N =*** **108**
Mean (SD)	53.1 (13.7)
Range	22–84
**Educational level**	***N =*** **99**
Mean in years (SD)	14.6 (3.61)
**Age at diagnosis of first MDE**	***N =*** **58**
Mean (SD)	40.6 (15.6)
Range	18–83
**Number of prior MDEs**	***N =*** **56**
Mean (SD)	3.96 (3.37)
Range	1–20
**Number of prior treatment-resistant MDEs (≥ 2 ADs)**	***N =*** **54**
Mean (SD)	1.52 (1.00)
Range	0–7
Characteristics of the current MDE^*^	***N =*** **56**
Mild **(N, %)**	1 (1.8%)
Moderate **(N, %)**	10 (17.9%)
Severe without PF **(N, %)**	36 (64.3%)
Severe with congruent PFs **(N, %)**	8 (14.3%)
Severe with incongruent PFs **(N, %)**	1 (1.8%)
**Other characteristics of the current MDE**	***N =*** **56**
MDE with atypical characteristics **(N, %)**	1 (1.8%)
MDE with melancholic symptoms **(N, %)**	8 (14.3%)
Non-melancholic, atypical, catatonic MDE	47 (83.9%)
**Duration of the current MDE**	***N =*** **56**
Total duration ≥ 2 years (chronicity)	30 (53.6%)
Time to introduction of AD	***N =*** **41**
In weeks, median, SD	4 (80.8)
Range	0–364
**MADRS**	***N =*** **108**
Mean (SD)	27.9 (6.71)
Range	13–45
**QIDS**	***N =*** **107**
Mean (SD)	16.0 (4.83)
Range	4–26
**BAS**	***N =*** **101**
Mean (SD)	15.6 (7.47)
Range	0–36
**STAI-A**	***N =*** **107**
Mean (SD)	50.6 (10.1)
Range	27–72
**Rosenberg**	***N =*** **99**
Mean (SD)	21.5 (5.87)
Range	10–36
**CTQ**	***N =*** **106**
Mean (SD)	41.9 (12.6)
Range	25–88
**Sexual abuse**	***N =*** **106**
Mean (SD)	6.09 (3.24)
Range	5–24
**Physical abuse**	***N =*** **106**
Mean (SD)	6.20 (2.30)
Range	5-−20
**Emotional abuse**	***N =*** **106**
Mean (SD)	9.43 (4.39)
Range	5–22
**Physical neglect**	***N =*** **106**
Mean (SD)	7.27 (2.64)
Range	5–20
**Emotional neglect**	***N =*** **106**
Mean (SD)	12.9 (4.94)
Range	5–25

*AD, Antidepressant; PF, Psychotic Features; SD, Standard Deviation; MDE, Major Depressive Episode; N, total number of participants*.

* *According to DSM-IV classifications*.

### Antidepressants and Therapeutic Strategies

Regarding the use of AD at V0 (see Table 3), one in every four patients received a SNRI (Duloxetine, Milnacipran or Venlafaxine), 20.4% a SSRI (Citalopram, Escitalopram, Fluoxetine, Paroxetine, Sertraline or Fluvoxamine), 17.6% a TCA (Amitriptyline, Amoxapine, Clomipramine, Dosulepin (Dothiepin), Doxepin, Imipramine or Maprotiline), 5.6% a NASSA (Mianserin or Mirtazapine), 3.7% a MAOI (Moclobemide, Iproniazid, Phenelzine or Tranylcypromine) and 2.8% “another AD” (Agomelatine, Bupropion, Vortioxetine or Tianeptine) while the remaining 24.1% received a combination of at least 2 distinct ADs.

**Table 3:** Concomitant antidepressant, potentiator and symptomatic treatments.

**A. Current treatment**		**B. Therapeutic strategies**	
**Antidepressant treatment (N, %)**	***N =* 108**	**Background treatment**	***N =* 108**
SSRI	22 (20.4%)	**SSRI (N, %)**	**22 (20.4%)**
SNRI	28 (25.9%)	Non-potentiated	12 (11.1%)
ATC	19 (17.6%)	With MS/AD	2 (1.9%)
NASSA	6 (5.6%)	With SGA	6 (5.6%)
MAOI	4 (3.7%)	With ≥ 1 *other* PT	1 (0.9%)
Other antidepressant	3 (2.8%)	With ≥ 2 PTs	1 (0.9%)
Combination of ≥ 2 ADs	26 (24.1%)	**SNRI (N, %)**	**28 (25.9%)**
**Potentiation treatment (N, %)**	***N =*** **108**	Non-potentiated	16 (14.8%)
None	53 (49.1%)	With Lithium	4 (3.7%)
Lithium	8 (7.4%)	With MS/AD	3 (2.8%)
MS/AD	10 (9.3%)	With SGA	3 (2.8%)
AP2G	30 (27.8%)	With ≥ 1 *other* PT	1 (0.9%)
Other potentiator	2 (1.9%)	With ≥ 2 PTs	1 (0.9%)
Association of ≥ 2 PT	5 (4.6%)	**TCA (N, %)**	**19 (17.6%)**
**Symptomatic treatment (N, %)**	***N =*** **108**	Non-potentiated	10 (9.3%)
None	48 (44.4%)	With MS/AD	1 (0.9%)
ANTI-H1	2 (1.9%)	With SGA	8 (7.4%)
Antipsychotic	3 (2.8%)	**NASSA (N, %)**	**6 (5.6%)**
Benzodiazepine/Z-drug	35 (32.4%)	Non-potentiated	1 (0.9%)
Combination of BZD and AH1	4 (3.7%)	With Lithium	3 (2.8%)
Combination of BZD and AP	5 (4.6%)	With SGA	2 (1.9%)
*Non-specified* treatment	11 (10.2%)	**MAOI (N, %)**	**4 (3.7%)**
		Non-potentiated	3 (2.8%)
		With SGA	1 (0.9%)
		**Other AD (N, %)**	**3 (2.8%)**
		Non-potentiated	1 (0.9%)
		With MS/AD	1 (0.9%)
		With SGA	1 (0.9%)
		**Combination of ≥ 2 ADs (N, %)**	**26 (24.1%)**
		Non-potentiated	10 (9.3%)
		With Lithium	1 (0.9%)
		With MS/AD	3 (2.8%)
		With SGA	9 (8.3%)
		With ≥ 2 PTs	3 (2.8%)

*AD, Antidepressant; ANTI-H1, AH1: H1 antihistamine; AP, Antipsychotic; SGA, Second-generation antipsychotic; TCA, Tricyclic antidepressant; BZD, Benzodiazepine or Z-drug; MAOI, Monoamine oxidase inhibitor; SNRI, Serotonin norepinephrine reuptake inhibitor; SSRI, Selective serotonin reuptake inhibitor; NASSA, α2 adrenoceptor antagonist; MS/AD, Mood stabilizer or antiepileptic drug; PT, Potentiator treatment*.

Furthermore, half of the patients were concomitantly treated with a “potentiator,” which was a second-generation antipsychotic (SGA) (Amisulpride, Aripiprazole, Clozapine, Olanzapine, Quetiapine or Risperidone) in 27.8% of cases, a mood stabilizer or antiepileptic drug (MS/AD) (Lamotrigine, Pregabalin, Topiramate, Valproate or Valpromide) in 9.3% of cases, Lithium in 7.4% of cases, “another potentiator” (Buspirone, Methylphenidate, Modafinil or Pramipexole) in 1.9% of cases and a combination of at least 2 of the previous molecules (including thyroid hormones) in the remaining 4.6% of cases. The detail of the potentiators for each class of ADs is also presented in Table 3.

In addition, 55.6% of the patients received a concomitant symptomatic treatment, namely a benzodiazepine or Z-drug (BZD) in 32.4% of cases, an antipsychotic (AP) in 2.8% of cases, an H1-antihistamine (ANTI-H1) in 1.9% of cases, a combination of BZD and AP in 4.6% of cases and a combination of BZD and ANTI-H1 in 3.7% of cases (with data being incomplete for 10.2% of cases).

### Side Effects

The mean score on the side-effects scale (PRISM) was 13.3 points among the 108 participants with the theoretical total score ranging from 0 to 31. Four and seven of the 32 SEs were reported in more than 80% and 60% of cases, respectively. As indicated in Table 4, the most commonly reported SEs were concentration impairment, asthenia, perceived loss of energy, anxiety, xerostomia, general malaise, reduced sex drive, and erectile dysfunction.

**Table 4:** The most common side effects.

**Rank**	**Side effect**	**Amongst N**	**Occurrence (*N*, %)**	**SE score (mean, SD)**
1	Other disorders: concentration	*N =* 108	98 (90.7%)	0.907 (0.291)
2	Other disorders: asthenia		94 (87.1%)	0.870 (0.337)
3	Other disorders: loss of energy		93 (86.1%)	0.861 (0.347)
4	Other disorders: anxiety		90 (83.3%)	0.833 (0.374)
5	Gastrointestinal disorders: dry mouth		74 (68.5%)	0.685 (0.467)
6	Other disorders: general malaise		73 (67.6%)	0.676 (0.470)
7	Sexual function: reduced sex drive		69 (63.9%)	0.639 (0.483)
8	Sexual function: erectile dysfunction	*N =* 41 (M)	25 (61.0%)	0.610 (0.494)

*SD, Standard Deviation; M, Males; MeaN, mean; N, total number of participants*.

Importantly, no difference was found among the 7 treatment groups (i.e., SSRI, SNRI, TCA, NASSA, MAOI, other ADs, and combination of ADs) regarding the occurrence of SEs, except for an over-reporting of tremors in patients receiving a combination of ADs compared to subjects receiving serotonergic drugs (MD = −36390; SE = 0.113; *p* = 0.014).

### Socio-Demographic Variables

#### Age

No linear relationship was found between age and total SE score. However, patients aged 40 to 60 years presented a higher total SE score compared to that in the older age group (Mean Difference (MD) = 4.00; SE = 1.11; *p* = 0.001). A similar result was observed among the domains of SEs, with more “other SEs” reported by patients aged 40 to 60 years as compared to older subjects (MD = 1.39; SE = 0.337; *p* < 0.001). However, with regard to the 32-item scale, only the occurrence of headaches was significantly associated with the age categories, with an over-reporting of headaches in the 40–60 age group compared to older subjects (MD = 0.370; SE = 0.101; *p* = 0.001).

#### Sex

The patient's sex was not associated with any significant difference in the total SE score. A significant sex effect was, however, observed in the different domains explored, with sexual dysfunctions more frequently reported by men (MD = 1.06; SE = 0.201; *p* < 0.001).

#### Level of Education

Conversely, the level of education was not associated with any significant difference in the occurrence of SEs.

### Psychopathological Status and Psychometric Data

#### Depression

The intensity of depressive symptomatology assessed using the MADRS score was positively correlated with the overall occurrence of SEs, which remained after adjustment for age and sex (β = 0.2291; *p* = 0.017), and more incidentally with impaired concentration (β = 0.400; *p* < 0.001). In particular, patients with a higher MADRS score reported more headaches (β = 0.260; *p* = 0.007), xeroderma (β = 0.3056; *p* = 0.002) general malaise (β = 0.28577; *p* = 0.003), asthenia (β = 0.3537; *p* < 0.001), and a loss of energy (β = 0.3788; *p* < 0.001). A similar effect was also recorded in the total SE score for the self-rated depressive severity using the QIDS (β = 0.464; *p* < 0.001), which was associated with a significant increase in SEs in the digestive (β = 0.2299; *p* = 0.019), cardiovascular (β = 0.22793; *p* = 0.004), cutaneous (β = 0.3392; *p* < 0.001), neurological (β = 0.2820; *p* = 0.004), and *other* systems (β = 0.4141; *p* < 0.001). The duration of current episode was not associated with occurrence of SEs (β =- 0.156; *p* = 0.33; ANOVA: <1 month; 1–6 months; 6–24 months; >24 months; *p* = 0,644).

#### Anxiety

The increase in the total score on the BAS was significantly associated to that in the overall SE score (β = 0.4409; *p* < 0.001) indicating an increase in cardiovascular (β = 0.3665; *p* < 0.001), cutaneous (β = 0.2904; *p* = 0.006), sensory (β = 0.4775; *p* < 0.001) and sleep (β = 0.2360; *p* = 0.024) symptoms as well as in the *other* SEs (β = 0.03807; *p* < 0.001). Among these, an over-representation of nausea and vomiting (β = 0.2493; *p* = 0.019), palpitations (β = 0.35790; *p* < 0.001), vertigo (β = 0.2380; *p* = 0.025), chest pain (β = 0.2174; *p* = 0.045), headache (β = 0.30725; *p* = 0.004), pruritus (β = 0.3101; *p* = 0.004), blurred vision (β = 0.3439; *p* = 0.001), tinnitus (β = 0.3528; *p* = 0.001), sleep disorders (difficulties falling asleep) (β = 0.255; *p* = 0.015) as well as anxiety (β = 0.36585; *p* < 0.001), general malaise (β = 0.3124; *p* = 0.003), agitation (β = 0.3625; *p* < 0.001) and asthenia (β = 0.2792; *p* = 0.007) were documented in the most anxious patients. Similarly, a higher level of state-anxiety measured on the STAI was significantly associated with increased reporting of overall SEs (β = 0.3152; p 0.002) and of non-specific SEs in particular (β = 0.08237 ± 0.0149; β = 0.4990; *p* < 0.001), with an increase in anxiety (β = 0.4256; *p* < 0.001), general malaise (β = 0.3237; *p* = 0.001), agitation (β = 0.390; *p* < 0.001), and asthenia (β = 0.2878; *p* = 0.004), along with a loss of energy (β = 0.2497; *p* = 0.015).

#### Self-Esteem

The increase in the Rosenberg scale score was negatively correlated with the occurrence of SEs. In other words, a decrease in the self-esteem score was associated with an increase in the total SE score (β = −0.2852; *p* = 0.020) and in non-specific SEs (β =−0.36448; *p* = 0.003) including anxiety (β = −0.3886; *p* = 0.001), general malaise (β =−0.2569; *p* = 0.040) and asthenia (β = −0.4235; *p* < 0.001).

#### Suicidality

The measure of suicidal ideation and related intentionality (MSI) showed a positive correlation between the overall occurrence of SEs (β = 0.2415; *p* = 0.023), with an increase in digestive (β = 0.3077; *p* = 0.004), cardiovascular (β = 0.2254; *p* = 0.039) and cutaneous symptoms (β = 0.2216; *p* = 0.041).

#### Functioning

No significant association was found between the overall functioning on the GAF and the overall assessment of the SEs, although a negative correlation was highlighted between the quality of functioning and the reporting of digestive disorders (β =−0.2211; *p* = 0.046), with increased complaints of xerostomia in patients with a less satisfactory level of functioning (β = −0.3332; *p* = 0.003).

### Other Clinical and Environmental Characteristics

#### Childhood Trauma

Several components of the CTQ—namely the overall score, as well as 3 of the 5 defined subscores—were significantly associated with the reporting of SEs, particularly in the sexual and emotional domains. There was a positive correlation between the total score on the CTQ and the overall occurrence of SEs (β = 0.302; *p* = 0.002), but also with cutaneous symptoms (β = 0.2163; *p* = 0.028), urogenital symptoms (β = 0.2692; *p* = 0.006) and *other* SEs (β = 0.3229; *p* < 0.001). In terms of the different items, trauma exposure was associated with decreased sex drive (β = 0.3142; *p* = 0.001) and weight gain (β = 0.3192; *p* = 0.001). Likewise, a history of sexual abuse was correlated with the overall SE score (β = 0.211; *p* = 0.029) and a similar finding was observed at the emotional level, with a correlation between a history of abuse and the occurrence of SEs both on a global scale (β = 0.3360; *p* < 0.001) and in different domains regarding cardiovascular (β = 0.2250; *p* = 0.02), cutaneous (β = 0.1984; *p* = 0.045), sensory (β = 0.25879; *p* = 0.010), sleep (β = 0.2034; *p* = 0.032) and *other* SEs (β = 0.2966; *p* = 0.002). Despite the absence of correlation with the total SE score, an association was found between a history of emotional neglect and non-specific SEs (β = 0.2625; *p* = 0.007). However, it should be noted that there were no significant relationships between neither physical abuse nor physical neglect and occurrence of SEs.

#### Lifestyle and Consumer Habits

The body mass index and the WHO BMI categories did not appear to be significantly associated with the occurrence of SEs. Similarly, the patient smoking status (current smoker, ex-smoker or non-smoker) as well as the quantification of tobacco consumption in pack-years did not highlight any significant association with the reporting of SEs.

## Discussion

To our knowledge, this study is the first observational study to address the determinants of the occurrence of SEs in a cohort of TRD patients receiving ADs. The occurrence of SEs under AD in patients with TRD seemed to be influenced by the age and by the sex of patients. The SE scores showed a positive correlation with the intensity of anxious, depressive and suicidal symptoms and a history of childhood abuse—in particular sexual (abuse) and emotional (abuse and negligence). Conversely, the reporting of SEs was negatively related with self-esteem, and with the global assessment of functioning.

The first determinants of SEs identified stemmed from socio-demographic parameters. In older populations, MDD is added to the physiological changes associated with aging, in often polypathological and polymedicated subjects (26), who are well-recognized to be more sensitive to the pharmacotoxicity of psychotropic drugs (27) and therefore prone to SEs (28). Paradoxically, in our study, the reporting of SEs was lower in those patients over 60 years than in the younger subjects for the overall SE score, the non-specific SEs score and the occurrence of headaches. These observations matched those within the cohort by Bet *et al*. explaining this discrepancy—for class-specific SEs—by the senescence of the serotoninergic system that could account for reduced SE occurrences in aged depressed patients (17, 29). Interestingly, more specific SEs were identified in patients receiving ADs showing an increased risk of hyponatraemia, osteoporosis, or falls [53, 58] that seemed more prevalent in the elderly [59]. Since these specific SEs have not been investigated in our study, their existence could also explain the previous observations suggesting the influence of age, if not on the actual frequency of occurrence, at least on the *profile* of the SEs observed in patients receiving ADs.

In our study, influence of sex was also observed, which seemed to be limited—to a significant extent—to sexual dysfunctions, mainly in men. Despite a lack of robustness following corrections for multiple comparisons, greater loss of sex drive and orgasm disorders were reported by men, and erectile dysfunction normalized to the male population was among the 8 most frequent SEs in our sample. However, in the general population, the prevalence of sexual dysfunctions is higher in women (30–32). Therefore, female subjects would be less likely to report a difference under AD therapy compared to their standard state. At the same time, a study on AD-induced sexual disorders showed that post-menopausal women—probably over-represented in our study population regarding the mean age of the participants—considered treatment-induced sexual disorders as a “minor” problem that they “accepted” more easily than the other patients (33). Thus, this could explain the increase in sexual dysfunction reported more specifically by men in our study.

The clinical severity of the depressive symptomology was positively correlated with the overall SE score, while for the self-rated intensity of depression, an association was found with the occurrence of SEs in 5 categories as well as an over-reporting of general SEs (general malaise, fatigue, loss of energy) and more specific symptoms (xeroderma, headaches). These results are consistent with the findings in non-treatment-resistant MDD, with a higher number of SEs per subject described in more severe depression, regardless the classes of AD (17), focusing on tricyclic ADs (18), or even on Lithium in bipolar MDEs (34). Also, the anxiety level of the patients was positively correlated with the overall occurrence of SEs. These findings are in line with the observation that a higher level of “worry” was associated with a higher number of side effects and an earlier dropout of AD treatment (18), with a predictive value in non-remission in TRD patients (22). In the same way, while the contribution of low self-esteem to depressive symptoms and treatment prospects has already been highlighted (35), only limited data was available on the relationship between the level of self-esteem and the occurrence of SEs, that we have showed to be negatively corelated.

Regarding environmental factors, even if several authors have shown early life stress to be associated with a poorest response to antidepressant therapy for MDD (23), the relationship between childhood trauma and SEs in patients receiving ADs had not yet been investigated. In our study, a previous history of childhood trauma was associated with the onset of SEs in terms of overall occurrence, in different domains, and for two items, i.e., loss of sex drive and weight gain. Remarkably, since a history of abuse has been identified as a risk factor for obesity (36) and eating disorders (37), particular attention needs to be paid to these patients when prescribing ADs.

Concerning antidepressant treatments, no difference was found between the different classes of AD, except for the reporting of tremors in subjects receiving combination therapy in comparison to serotonin-acting monotherapies. In this respect, a meta-analysis comparing the occurrence of SEs between SSRIs and TCAs in patients with non-treatment-resistant depression identified different SE profiles depending on the pharmacological classes (15), whereas Bet *et al*. observed a greater number of SEs with TCAs than with SSRIs (17) and distinct SE profiles between the different groups of ADs. Yet, these findings were not observed in our study. This discrepancy could be explained by the differences in the populations studied, but also by the systematic exploration of the 9 domains of SEs—grouped by functional organs rather than by pharmacological classes—and of the 32 items, with a risk of diluting the effect of the SEs most likely to have class-related specificities by the more transversal SEs.

In addition, our study was carried out under real practice conditions and was not influenced by the timing of the investigations. SEs were not assessed at a given time point after AD medication was initiated but when patients were enrolled into the cohort, while not specifically targeting the introduction of the treatment studied. This assumption would be in line with previous observations leading to consider that the greatest number of SEs is expected after the beginning of the treatment, before decreasing with extended use (18) or with the reintroduction of an already used AD (17). Likewise, since our study patients had already undergone unsuccessful treatment attempts, it can be expected that ADs that had already been proposed and poorly tolerated were not considered once again. Thus, as patients were not randomly assigned to the treatment groups, the proposed therapeutic adjustments—although clinically relevant—probably limited the risk of the occurrence and therefore the *detection* of SEs in our study, by excluding the molecules most likely to cause them.

### Strengths of This Study

One of the strengths of our work was that we carried out a multi-center study using a naturalistic approach. Also, we used a specific and valid clinical instrument in order to perform a reliable assessment of SEs. Although of moderate size, our sample included 108 patients receiving specialized tertiary care. Despite the absence of a control group, the multi-center recruitment carried out in all 13 Expert Centers and the choice of a naturalistic care approach (i.e., depending on the psychiatrist's evaluation for each patient), ensures that medical practice was reflected across the country and under real conditions, from a daily clinical and patient-centered perspective.

The score extracted for the SEs, based on the data collected using the PRISE-M scale and re-coded in terms of occurrence, also appears to be of considerable methodological interest. Firstly, the relevance of scores without the level of perceived symptomatic severity can limit potential bias relating to the patient's subjectivity (inter-individual differences) and therefore reinforces, through the deletion of a confounding factor, the relationships between the variables examined and the rate of SEs.

Similarly, using the PRISE-M scale with a restricted timeframe overcomes, another limit identified in the Dutch study (17) by minimizing the recall bias thereby leading to contribute to the standardization of assessment methods and the reproducibility of findings.

In addition, the few studies addressing the determinants of SEs in patients receiving ADs appear to be restricted to a relatively small number of symptoms (17, 38). On the contrary, the systematic confrontation of each variable with a score for the overall occurrence of SEs, the 9 defined areas, and the 32 explored items added a multidimensional scale to our analyses.

### Limitations

Our study had some limitations. First, the use of the database of the Expert Centers collected before determining the inclusion parameters for our study, led us to exclude nearly two-thirds of the eligible subjects (therapeutic window at t0, missing treatment-related data, missing side-effects-related data), thereby limiting the size of our study sample. Second, besides—and as developed earlier—SEs were not assessed at a defined time point after the AD was initiated among the remaining patients, and the ADs that had already been prescribed and poorly tolerated were probably “avoided” by their attending psychiatrist, which once again limits the likelihood of occurrence and detection of SEs at the time of inclusion. Moreover, SEs were not assessed with regards to the duration of antidepressant treatments. Third, some pharmacological classes were heterogeneous focusing on mechanisms of action. Four, the rating scale selected by the Expert Centers itself raises the question of the symptoms considered as direct effects of the ADs, whose etiology still remain to be confirmed. Although precautions were taken during prior processing of the database (inclusion limited to information relating to ongoing treatment at baseline or interrupted <1 week before the period covered by the questionnaire)—the possible overlapping of certain items and depressive symptoms remains a legitimate question (39, 40). Thus, in order to reduce the associated attribution bias, the results of the various analyses were therefore systematically corrected by the hetero-assessed depressive symptomology (MADRS). Then, although the study of Bet et al. was conducted on patients with a standard “depressive disorder and/or an anxiety disorder” (17) while our study only focused on patients with TRD, these differences at baseline do not guarantee the absence of comorbid anxiety disorder or “anxious distress,” frequently associated with depression (41). Conversely, since most of the symptoms defining generalized anxiety disorder or panic attack (41) are found in the PRISE-M scale and labeled “SEs,” it should be noted that our findings also raise the question of the etiology of the symptoms assessed by the PRISE-M scale, i.e., the possible overlapping of SEs and comorbid anxiety symptoms.

Functional alterations and psychopathological characteristics assessed by the various psychometric scales could, in turn, represent a predisposing factor for the onset of SEs as well as a consequence of SEs. For example, suicidal ideations, whose relationship with ADs are still the subject of controversy (42), could be equally considered a predisposing factor for the occurrence of SEs, a SE *per se*, or a direct consequence of the SEs. Similarly, an increase in dry mouth symptoms was associated with a decrease in the GAF, where the alteration in the second variable would *intuitively* result from the changes in the first one. For this reason, the notion of association considered in our study seems reasonably preferable to the hypothesis of a one-way causal relationship.

## Conclusion

To our knowledge, this is a first observational study focused on the factors strongly associated with the occurrence of SEs under ADs in a cohort of patients with TRD by analyzing clinical and pharmacological features along with sociodemographic and environmental characteristics, thereby resulting in an extensive overview of this issue. The choice of routinely available parameters and the proposal of a naturalistic approach ensured an ecological framework, allowing these results to be transposed into current practice. Beyond the epidemiological and descriptive approach, the stakes surrounding SEs touch the concept of drug compliance while the required pre-therapeutic reflection appears in line with the dynamics of individualized medicine. This patient-centered approach includes integrated pharmacological care in accordance with clinical practice guidelines and designed to promote the continuation of antidepressant treatments by reducing the number of early interruptions related to SEs or uncomfortable switches and thus, by definition, preventing the further development of *treatment resistance*.

## Data Availability Statement

The raw data supporting the conclusions of this article will be made available by the authors, without undue reservation.

## Ethics Statement

The authors assert that all procedures contributing to this work comply with the ethical standards of the relevant national and institutional committees on human experimentation and with the Helsinki Declaration of 1975, as revised in 2008. The patients/participants provided their written informed consent to participate in this study.

## Author Contributions

All authors were involved in the identification and selection of patients, in the clinical assessment, and co-wrote the paper. AL, AY, WE-H, and BA were also involved in choosing the data set for this project and the statistical analysis. All authors contributed to the article and approved the submitted version.

## Funding

This research was funded by the FondaMental Foundation, Institut National de la Santé et de la Recherche Médicale (INSERM), AP-HP, Astra Zeneca, and by the Investissements d'Avenir programme managed by the ANR under reference ANR-11-IDEX-0004-02. This funding source had no role in the study design, data collection, analysis, preparation of the manuscript, or decision to submit the manuscript for publication.

## Conflict of Interest

AY received speaker's honoraria from AstraZeneca, Janssen, Lundbeck, Otsuka, Servier and carried out clinical studies in relation to the development of medicine Janssen and Lundbeck medicine unrelated to this work. J-BG received a speaker's honorarium from Servier. P-ML received grants, honoraria, and consulting fees from Allergan, Gedeon Richter, Janssen- Cilag, Lundbeck, Otsuka, Recordati, Sanofi-Aventis, and Teva. RRi received a speaker's honorarium from Janssen Cilag. FS received honoraria from Otsuka. EH acted in advisory capacities, carried out clinical studies in relation to the development of a medicine, received personal researches, studies, or travel allowance, gave presentations at meetings, and received remuneration for input from the following pharmaceutical organizations: AstraZeneca, BMS, Cellgene, Euthérapie-Servier, Janssen, Elli Lilly, Lundbeck, LivaNova, Otsuka, Pfizer, Sanofi. WE-H received speaker's honoraria from Chugai, Eisai, Lundbeck, Janssen-Cilag, Otsuka, and UCB unrelated to this work. BA received speaker's honoraria and/or a travel allowance from Lundbeck, Sanofi, Janssen-Cilag, and Eli Lilly. He has served on the advisory board of Janssen-Cilag. BE received honoraria for consulting activities for Sanofi. The remaining authors declare that the research was conducted in the absence of any commercial or financial relationships that could be construed as a potential conflict of interest.

## Publisher's Note

All claims expressed in this article are solely those of the authors and do not necessarily represent those of their affiliated organizations, or those of the publisher, the editors and the reviewers. Any product that may be evaluated in this article, or claim that may be made by its manufacturer, is not guaranteed or endorsed by the publisher.

## Abbreviations

AD: Antidepressant
SE: Side effect
ANOVA: Analysis Of Variance
ANCOVA: Analysis Of Covariance
ATHF: Antidepressant Treatment History Form
BAS: (Tyrer) Brief Anxiety Scale
BMI: Body Mass Index
BZD: Benzodiazepines
CRP: C-Reactive Protein
CSSRS: Columbia Suicide Severity Rating Scale
CTQ: Child Trauma Questionnaire
DSM: Diagnostic and Statistical Manual of Mental Disorders
GAF: Global Assessment of Functioning
ANTI-H1: H1 Antihistamine
MADRS: Montgomery-Åsberg Depression Rating Scale
MAOIs: Monoamine Oxidase Inhibitor
MDD: Major Depressive Disorder
MDE: Major Depressive Episode
MS/AD: Mood Stabilizer or Anti-epileptic Drug
NASSA: Noradrenergic And Specific Serotoninergic Antidepressant
PF: Psychotic Features
PRISE-M: Patient-Rated Inventory of Side Effects Modified
QIDS: Quick Inventory of Depressive Symptomatology
SGA: Second Generation (or atypical) Antipsychotic
SNRI: Serotonin–Norepinephrine Re-uptake Inhibitor
SSRI: Selective Serotonin Re-uptake Inhibitors
STAI-YA: State-Trait Anxiety Inventory, YA form
TCA: Tricyclic Antidepressant
TRD: Treatment-Resistant Depression.
